# Exploring dentist’s engagement with antibiotic stewardship programs: a multicenter qualitative study in Pakistan

**DOI:** 10.3205/dgkh000586

**Published:** 2025-09-23

**Authors:** Muhammad Hassan, Muhammad Zaeem Ihsan, Abdul Sami, Mehvish Sajjad, Seema Shafeeq, Ahmed Talal, Ayesha Zahid, Arfa Tariq, Moghees A. Baig

**Affiliations:** 1 Science of Dental Materials, University College of Dentistry, University of Lahore, Lahore, Pakistan; 2 Department of Community and Preventive Dentistry, University College of Dentistry, University of Lahore, Lahore, Pakistan; 3 Faculty of Education, University of Ottawa, Ontario, Canada; 4 University College of Dentistry, University of Lahore, Lahore, Pakistan

**Keywords:** antibiotic stewardship, qualitative research, antibiotic resistance

## Abstract

**Introduction:**

Antibiotic resistance is a growing threat to public health. Improper use of antibiotics can potentially lead to previously curable infections becoming unmanageable. Rampant unnecessary use of antibiotics is a serious problem within developing countries like Pakistan, and dental practices can often be traced as a major cause. Through the widespread implementation of antibiotic stewardship programs (ASP), medical and dental practitioners can be guided to appropriate prescribing practices and better patient education.

**Objective:**

To gauge current antimicrobial prescribing practices of dentists and their engagement with ASPs.

**Methods:**

13 dental practitioners were interviewed using a semi-structured interview template. Interviews were recorded, transcribed, and subjected to thematic analysis. The subjects were selected from various dental hospitals within Punjab, using a convenient sampling approach. The sample size was based on the point of saturation of emerging themes.

**Results:**

Many factors were highlighted as causative for the current state of misinformation regarding antibiotic prescription practices among dental practitioners and patients. Awareness regarding ASP was severely lacking; however, most participants showed a positive perception regarding ASP and their impact. Institutional support and the need for implementation of such programs within the educational curriculum were noted as important steps for ASP implementation within dental hospitals of Pakistan.

**Conclusion:**

This study found that knowledge regarding ASP was insufficient among dental practitioners, owing to a lack of institutional policies and awareness among the practitioners. The outcome of the lack of these programs and misinformation among both patients and dentists is widely contributing to the current state of antibiotic resistance in Pakistan.

## Zusammenfassung

**Einleitung:**

Die Antibiotikaresistenz stellt eine wachsende Bedrohung für die öffentliche Gesundheit dar. Der unsachgemäße Einsatz von Antibiotika kann dazu führen, dass zuvor heilbare Infektionen unbeherrschbar werden. Der übermäßige, unnötige Einsatz von Antibiotika ist in Entwicklungsländern wie Pakistan ein ernstes Problem, und die zahnärztliche Praxis kann oft als eine der Hauptursachen angesehen werden. Durch die flächendeckende Einführung von Antibiotic-Stewardship-Programmen (ASP) können Ärzte und Zahnärzte zu einer angemessenen Verschreibungspraxis und einer besseren Patientenaufklärung angeleitet werden.

**Zielsetzung:**

Beurteilung der derzeitigen Verschreibungspraxis von antimikrobiellen Mitteln durch Zahnärzte und deren Engagement für ASP.

**Methode:**

13 Zahnärzte wurden anhand einer halbstrukturierten Interviewvorlage befragt. Die Interviews wurden aufgezeichnet, transkribiert und thematisch analysiert. Die Probanden wurden aus verschiedenen Zahnkliniken im Punjab nach dem Prinzip der Zufallsstichprobe ausgewählt. Die Stichprobengröße wurde auf der Grundlage des Sättigungsgrades der sich ergebenden Themen festgelegt.

**Ergebnisse:**

Viele Faktoren wurden als Ursache für den gegenwärtigen Zustand der Fehlinformation in Bezug auf die Verschreibungspraxis von Antibiotika unter Zahnärzten und Patienten hervorgehoben. Das Bewusstsein für ASP war sehr schwach ausgeprägt; die meisten Teilnehmer zeigten jedoch eine positive Einstellung zu ASP und deren Auswirkungen. Die institutionelle Unterstützung und die Notwendigkeit der Implementierung solcher Programme in den Lehrplan wurden als wichtige Schritte für die Implementierung von ASP in pakistanischen Zahnkliniken genannt.

**Schlussfolgerung:**

Die Studie ergab, dass das Wissen über ASP bei Zahnärzten unzureichend ist, was auf fehlende institutionelle Maßnahmen und mangelndes Bewusstsein bei den Ärzten zurückzuführen ist. Das Fehlen dieser Programme und die Fehlinformation sowohl der Patienten als auch der Zahnärzte tragen in hohem Maße zur derzeitigen Situation der Antibiotikaresistenz in Pakistan bei.

## Introduction

The introduction of Antibiotics in 1928 revolutionized the world of medicine and dentistry in terms of their effectiveness in controlling different bacterial infections, hence they were termed as miracle drugs [1]. According to a survey, 50% of hospitalized patients in the USA are taking antibiotics as a drug to treat different diseases [2].

Antibiotic consumption increased drastically by 65% during the period 2000–2015 due to the overuse of antibiotics without prescriptions, globally [3]. In 2015, the highest consumption of antibiotics was reported in India, China, and Pakistan [3]. Due to excessive use of antibiotics, drug-resistant strains of bacteria are spreading creeping pandemic [4]. The major reason is that people in underdeveloped countries don’t have the knowledge to use the antibiotics appropriately, and they are using them without any prescription [5].

With the discovery of penicillin, antibiotics became a critical part of global health, including cancer chemotherapy and advanced surgical procedures. Antimicrobial agents are not like other drugs. They are unique in that both the individual patient and the broader society bear the consequences of their use with each prescription. The antimicrobial effect that saves lives also exerts selective pressure on replicating bacteria, leading to the emergence of drug resistance [6].

Antibiotic resistance is a significant threat to global public health security. The prevalence of antibiotic resistance has assumed an alarming proportion the world over [7]. Antibiotic resistance accounts for significant causes of morbidity, mortality, and financial cost in the public health sector [8]. If left unchecked, this may lead to significant depreciation of antibiotic efficacy, leading to infections that may have been easily treated to become fatal [9].

There are certain guidelines in developed countries on the use of antibiotics, but unfortunately, such guidelines are yet to be implemented in Pakistan [10]. In this context, the advanced knowledge and awareness of health care professionals about the correct use of antibiotics could be a positive step in limiting antibiotic resistance while maintaining the effectiveness of antibiotics [11].

Antibacterial stewardship programs (ASPs) could be defined as ‘a coherent set of actions which promote using antimicrobials responsibly [12], [13]. ASPs allows for responsible and safe prescribing practice of antibiotics within healthcare settings, as well as guiding in the education of staff and identification of developing resistance among bacterial populations [14]. ASPs includes techniques to restrict the use of broad-spectrum antibiotics as well as to limit the use of antimicrobial agents to combat the resistance problems. It also covers certain guidelines to help healthcare professionals deal with proper antibiotic prescribing practices [15].

There is a literature gap regarding the exploration of the perspective of dentists in Pakistan about antimicrobial resistance, prescription practices, and their patients’ practices regarding antimicrobial use. The rationale of the study is to evaluate the clinical dental faculty’s knowledge and awareness of antibiotic stewardship programs and their implementation. Understanding the current perception of ASPs can allow for better implementation of such programs in Pakistan, as they have shown notable effectiveness when implemented in developing countries [16].

The current study aimed to identify the perspective of antimicrobial resistance and knowledge of ASPs among dentists in Pakistan. Being a developing country with a lack of legislation and oversight regarding prescribing practices, Pakistan has a high prevalence of multidrug resistant organisms (MDRO) [17]. Due to the OTC availability of many antibiotics, lack of healthcare facilities, and inadequate awareness among both the general public and medical professionals, Pakistan has developed a widespread issue with antimicrobial resistance [18].

## Methods

### Setting 

Dental Teaching Hospitals in Punjab.

### Study design 

Face-to-face interviews were conducted using a semi-structured questionnaire adapted from a study conducted to assess ASP awareness among medical doctors in Pakistan [4]. The interviews aimed to uncover themes centered on perception of antibiotic use and stewardship, antibiotic prescription practices, barriers to effective stewardship, and suggestions for enhancing ASP effectiveness. The themes included awareness of ASPs, challenges with antibiotic prescribing, encountering drug resistance, rational use of antibiotics, educational needs and recommendations, institutional support and guidelines and perceptions of ASPs’ potential impact.

Sample selection was done using purposive convenience sampling, in which a list of appropriate practitioners was made based on the previously mentioned criteria and keeping in view the specialties that more commonly prescribe antibiotics like maxillofacial surgical practice, operative dentistry/endodontics, and periodontology. Interviews were then conducted based on a convenience approach, where practitioners who had appropriate availability were chosen. Interviews were conducted in English as it was judged that the prospective participants all held an appropriate command over the language, since most had studied a majority English-based curriculum throughout their academic life.

Interviews were recorded and then transcribed for analysis by the interviewers themselves. Adherence to Consolidated Criteria for Reporting Qualitative Studies (COREQ) was kept [19].

## Results

### Participant characteristics 

The study included interviews of 13 dental professionals from various departments in dental hospitals across Pakistan, such as operative dentistry, oral and maxillofacial surgery, and oral medicine. The participants held positions ranging from assistant professor to head of department and professor, with clinical experience ranging from 4 to 20 years. The analysis of the interviews identified 7 major themes and 17 subthemes.

A lack of awareness about ASPs was evident among the participants. 11 out of 13 participants were unaware of ASPs or any related concepts. One participant remarked, “No, I am not aware of that”. This highlights the gap in exposure to ASPs concepts among the population of dentists in Pakistan. Despite the low awareness, once informed, participants were optimistic about the potential of ASPs, with one stating, “If these [programs] were common, they would bring about a change”.

The participants shared several challenges related to antibiotic prescribing. Participants noted that many patients self-medicate and demand antibiotics, often leading to inappropriate use. One interviewee commented, “Here in Pakistan which anyone can go to the pharmacy and get anything that they need over the counter. There are no rules and regulations”.

Some participants acknowledged an insufficient understanding of proper antibiotic use among dentists. One participant commented that “most of the dentists do not even know which antibiotics are broad spectrum and which antibiotics are narrow spectrum”.

A few participants highlighted the impact of pharmaceutical companies on prescribing practices, with aggressive marketing of higher-generation antibiotics influencing decisions.

The study revealed diverse perspectives on the rational use of antibiotics, due to limited diagnostic support and patient expectations. 9 out of 13 favored broad-spectrum antibiotics, while 8 out of 13 preferred narrow-spectrum antibiotics when diagnostics were available, or infections were less severe. 

However, despite misconceptions regarding broad-spectrum antibiotics as rational, nearly half believed antibiotics were unnecessary for routine dental cases like extractions or pulpitis. A majority (12 out of 13) identified inappropriate use as a major issue, attributing it to self-medication, over-the-counter availability, limited awareness of proper prescribing practices, and patient pressure.

Participants emphasized the importance of education to improve antibiotic use. Suggestions included workshops, seminars, and regular training sessions for faculty and students. 8 out of 13 participants explicitly advocated for integrating ASP into the dental curriculum. They emphasized that education at the undergraduate level is crucial for instilling proper prescribing practices and addressing antibiotic resistance. One participant proposed, “After every six months, there must be a workshop to update us on resistance patterns and appropriate prescribing”. Several participants stressed incorporating ASPs into the undergraduate curriculum to build a foundation for rational antibiotic use.

A significant gap in institutional support for antibiotic prescribing was highlighted. Only 1 out of 13 participants reported having formal guidelines in their institution. Most relied on personal judgment or informal practices, leading to inconsistent approaches to antibiotic use. Participants recommended developing and enforcing standardized guidelines at the institutional and national levels.

Despite low awareness, there was a consensus on the positive potential of ASPs; 10 participants believed that ASPs could bring significant improvements in prescribing practices. One stated, “These programs will create awareness about resistance and the proper usage and duration of antibiotics”.

**Figure 1:**
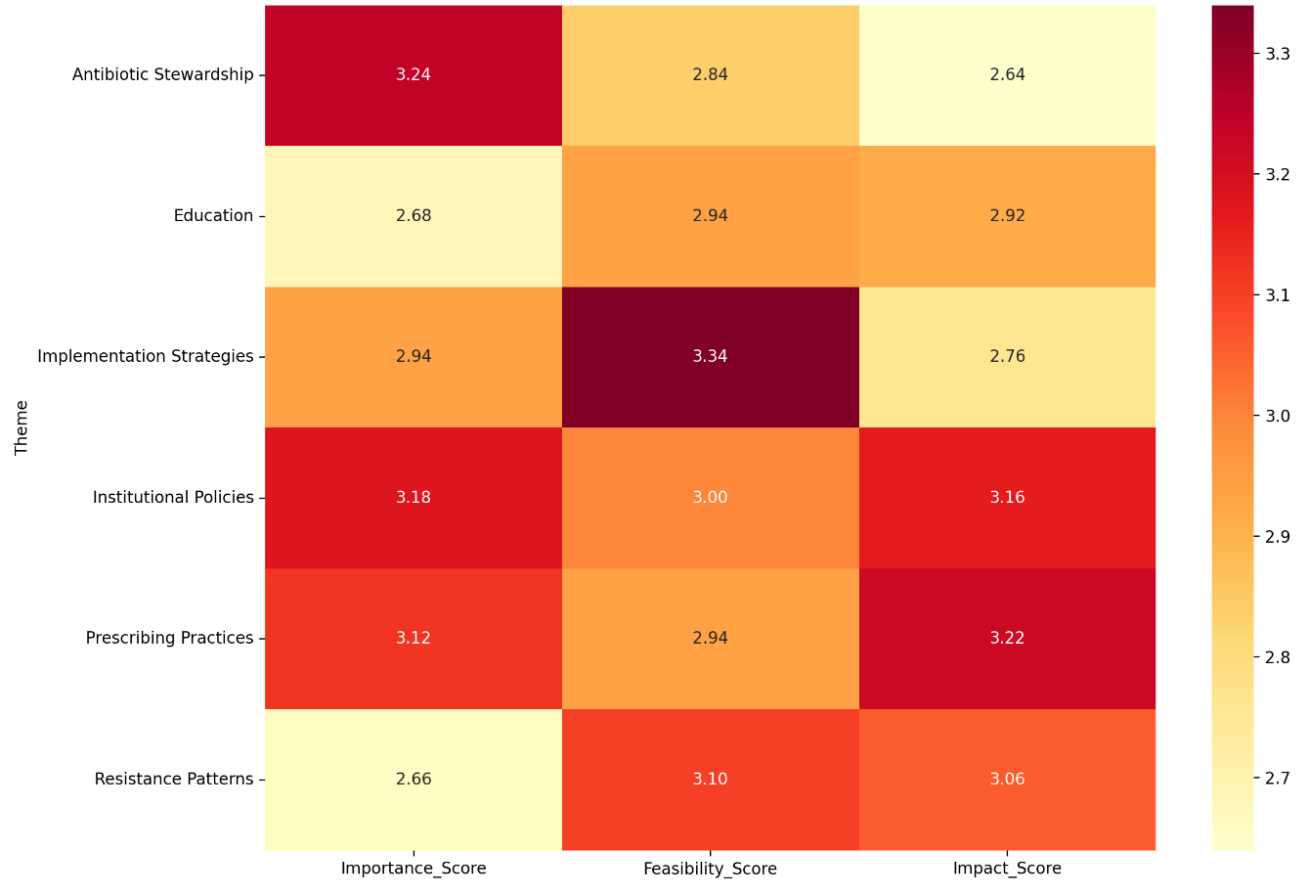
Heatmap visualizing mean scores across themes

Figure 1 (Fig. 1) shows a heatmap to allow more accurate visualization of the perception of different themes emerging from the interviews. On a scale from 1–5, the score variation ranges from 2.6 to 3.3, and scores are given for 3 distinct categories for 6 themes. The themes are scored on the perceived importance, feasibility of implementation, and perceived impact. The theme of Antibiotic stewardship is scored highest in importance, implementation strategies for combating antibiotic resistance are scored highest in feasibility, and the impact of appropriate prescribing practices is scored as the highest.

**Figure 2:**
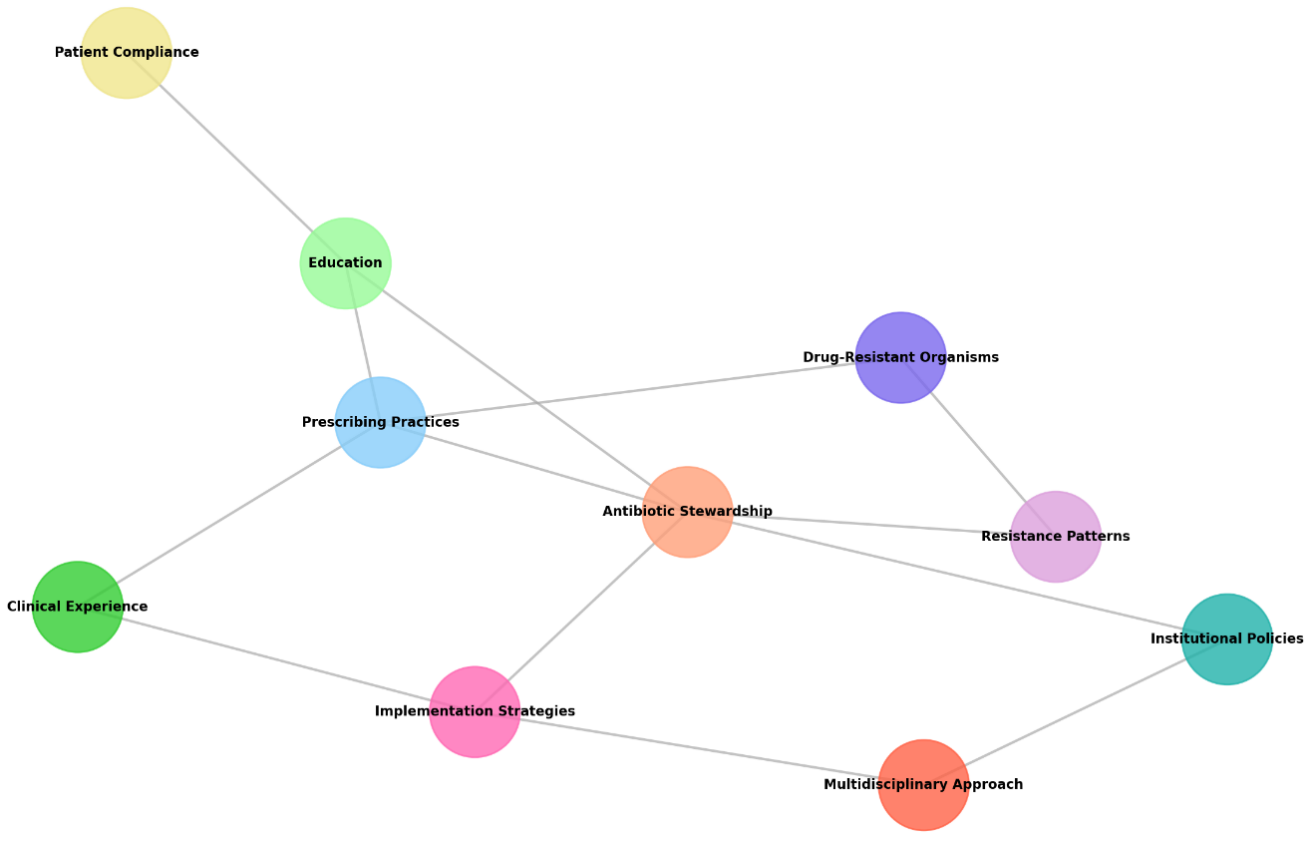
Network graph of themes identified in interviews

Figure 2 (Fig. 2) shows nodes representing identified themes from the conducted interviews. The size of the nodes correlates with their perceived importance, which is even across all nodes. The connecting lines between the nodes are a way to visualize the presence of connections and relations among these themes. Antibiotic Stewardship shows interconnectivity, both directly and indirectly, with most other nodes, as it is the core concept being investigated. 

## Discussion

Our study highlighted a lack of awareness of dentists with ASPs; only x out of 13 participants were aware of the term; these findings were similar to a study conducted among Medical doctors within Pakistan, which showed 17% participant awareness with ASPs [4]. While another study conducted on Pharmacy students in Pakistan showed that although many were aware of antimicrobial resistance, only 35% were familiar with ASPs [11]. These findings shed light on the fact that the healthcare system within Pakistan as a whole is not putting ample importance on the significance of ASPs. Similar to Pakistan hospitals in other South Asian countries, including India, Vietnam, and Bangladesh, lack formal ASPs [4], [20]. 

When asked about the prescription of broad-spectrum antibiotics, x out of 13 participants of the current study agreed that they prescribed them as a first line of defense. These findings show a larger trend in comparison with the practices of medical doctors in Pakistan, according to Atif et al. [4], who found 47% of respondents agreed to the use of broad-spectrum antibiotics as initial therapy. Participants in the current study cited a lack of diagnostic procedures, pressure from patients to achieve quicker relief, and the attempt to cover a wide range of pathogens as rationale for the frequent prescription of broad-spectrum antibiotics. 

12 out of 13 participants indicated an absence of formal guidelines for antibiotic prescription, with 10 participants showing a positive attitude towards the implementation of ASPs and establishing formal guidelines for antibiotic use. This shows a large inclination among practitioners to improve the current landscape of prescription practices, as adherence to formal guidelines has shown a shift to positive outcomes for patients [21], [22]. Atif et al. [4] and Hashemi et al. [23] in Pakistan and in India have shown a similar trend of prescribing practices based primarily on clinician discretion as opposed to present guidelines. 

Regarding experiences of participants with MDRO within their practices, 9 reported encountering such organisms in their practices. 8 mentioned not being aware of any specific organisms, whereas almost all participants mentioned that their hospitals did not perform blood cultures, so any identified organisms are based on existing knowledge and personal experience. This contrasts with Atif et al. [4], where most medical practitioners were able to identify specific microorganisms with reported resistance in their practices. This paints a picture of less rigorous reporting and investigating among dental hospitals in Pakistan regarding the burden of resistant organisms prevalent in their practices. 

In the study of Atif et al [4], medical doctors within Pakistan shared similar views on both the lack of available culture and sensitivity tests as well as patient influence on doctors. A study conducted in Canada and in US showed a lack of adherence to established guidelines and pressure from insurance companies as a driving factor for unnecessary antibiotic prescriptions among dentists [23]. 

### Limitations

Firstly, the study focused on dentists working within hospital setups, which allowed us to better gauge the prevalence of hospital guidelines concerning dental practices; however, the population of dentists working within dental hospitals is a minority compared to private practices. A study focusing on private practices as well would allow for a broader view on the engagement of dentists with ASPs within Pakistan at an individual level. Secondly, as data collection relied on convenience sampling, the reliability of the results in translating appropriately over Pakistan as a whole is limited [24].

## Conclusions

The current study highlights a significant disparity in knowledge of dental practitioners in Pakistan regarding ASPs. Lack of institutional policies, inadequate grasp of prescription practices among dentists, and widespread misconceptions among patients are leading causes of antimicrobial misuse within Pakistan. By embracing ASP practices as part of the educational curriculum for dental practitioners, implementing these programs within Dental hospitals in Pakistan, and scrutinizing antimicrobial misuse by government bodies, the disastrous effects of widespread antimicrobial resistance may be avoided.

## Notes

### Competing interests

The authors declare that they have no competing interests.

### Ethical approval 

Ethical Approval was received by the Institutional Review Board of the University College of Dentistry, The University of Lahore. Ref No: UCD/ERCA/24/444.

### Funding

None.

### Authors’ ORCIDs

- Hassan M: https://orcid.org/0000-0002-1753-7897
- Ihsan MZ: https://orcid.org/0009-0007-9246-5401
- Shafeeq S: https://orcid.org/0009-0007-8505-8947
